# Supplementary material for: Single-Cell RNA-Sequencing Reveals Epithelial Cell Signature of Multiple Subtypes in Chemically Induced Acute Lung Injury
Source: Int J Mol Sci. 2022 Dec 23;24(1):277. doi: 10.3390/ijms24010277 (PMC9820093; doi:10.3390/ijms24010277)

Supplemental Table S1. Sequences of the primer pairs used in the study.

Primers	Forward (5′–3′)	Reverse (5′–3′)
Actin	TTCAACGGCACAGTCAAGG	CTCAGCACCAGCATCACC
Sftpc	GAACACACAGACACCATCGC	ACCCTGAGATTGCCCGGAG
Sox9	CACATCTCTCCTAACGCCATCT	AAGCCTCAAGACTAGGGATGC
Epcam	ATTGCGGGGATTGTTGTCCT	ATCCTCCCCAGGTCTATCCG

Supplemental Table S2. Antibodies and their dilutions used for immunostaining.

Antibody	Dilution			Catalog number	Company
	IF	IHC	WB		
Sftpc	1:200	1:1000	1:1000	10774-1-AP	Proteintech
Sox9	1:200	1:2000	1:5000	ab185966	Abcam
Epcam			1:1000	ab213500	Abcam
TTF-1			1:2000	ab76013	Abcam
GAPDH			1:80000	SD0034	Simuwubio
Ki67	1:200			GTX1666	Abcam
PCNA	1:200			2586S	Abcam
IgG (H+L)			1:20000	SD0039	Simuwubio

IF, immunofluorescence; IHC, immunohistochemistry; WB, western blotting.

Supplemental Figure S1: High-quality BALF cells from the NC and Gas groups determined using scRNA-seq. A) tSNE plots of the batch effect between three different BALF samples. B) tSNE plots of the status of all cells when put above groups together with the reduced dimension cluster analysis of scRNA-seq data of the alveolar lavage cells and divided into 23 cell clusters. C) tSNE plots of the expression and distribution of marker genes in epithelial cells, macrophages, and macrophage proliferating cells. D) Dot plots of the expression of marker genes in all cells. (E) tSNE plot of the expression of canonical markers by each cell type. F) Violin plots of the expression of marker genes. G) Number of epithelial cells. H) Scatter plot of the number of genes, unique molecular identifiers (UMIs), and the percentage of mitochondrial genes in the samples. I) Epcam-labeled cells, and further classification of clusters 8, 18, 20, and 22, and identification as epithelial cells. J) tSNE plots of the top four maker genes of the above epithelial cells in each cluster. K) Feature genes were selected according to the average expression level of genes (≥0.1). L) The gradual change from dark blue to light blue indicates the change of pseudo-time. Pseudo-temporal trajectory of 11 subtype cells and pseudo-time trajectory of three different states.

Supplemental Figure S2: Annotation of pulmonary epithelial subtype cells based on the maker gene. A) Bar plot of the percentage of each cell cluster. B) Heatmap of the marker genes of each cluster of epithelial cells. C) Bubble plots of the marker genes in each cluster of epithelial cells. D) Scatter plot of classic marker genes in each epithelial cell type.

Supplemental Figure S3: Lung tissues were tested to assess the differences between the NC and Gas groups. A) Histopathological changes in the lung tissues of the NC and Gas groups. Rats were euthanized under ether anesthesia, and lungs from each experimental group were processed for histopathological evaluation. B) Lung injury score. A pathologist blindly scored each lung injury objectively according to a scoring system as previously reported [S1], and then calculated the mean of six lung sections using light microscopy (200× amplification; Olympus, Tokyo, Japan). Values are the means ± SEM (n = 6 in each group) of six independent experiments. C) qPCR of Epcam transcripts at different stages of lung development. D) Western blotting of lung lysates from phosgene-induced rats. Antibodies against Sftpc, Sox9, Epcam, TTF-1, and GAPDH were used. E) *Sftpc, Sox9, Epcam*, and *TTF*-1 transcript expression in ACEs of NC rats compared with that in the ACEs of Gas rats.

[S1] Gupta N, Su X, Popov B, Lee JW, Serikov V, Matthay MA. Intrapulmonary delivery of bone marrow-derived mesenchymal stem cells improves survival and attenuates endotoxin-induced acute lung injury in mice. J Immunol. 2007;179(3):1855–63.

Supplemental Figure S4: scRNA-seq reveals the differentially expressed genes (DEGs) in AECs from the NC and Gas groups. A) tSNE plots of the up- and downregulated DEGs in each cell cluster from the Gas (left) and NC (right) groups. KEGG pathway

analysis results of the B) citrate cycle, C) base excision repair, and D) mismatch repair. E) tSNE plots of the KEGG pathway analysis of signaling pathways regulating the pluripotency of stem cells.

Supplemental Figure S1

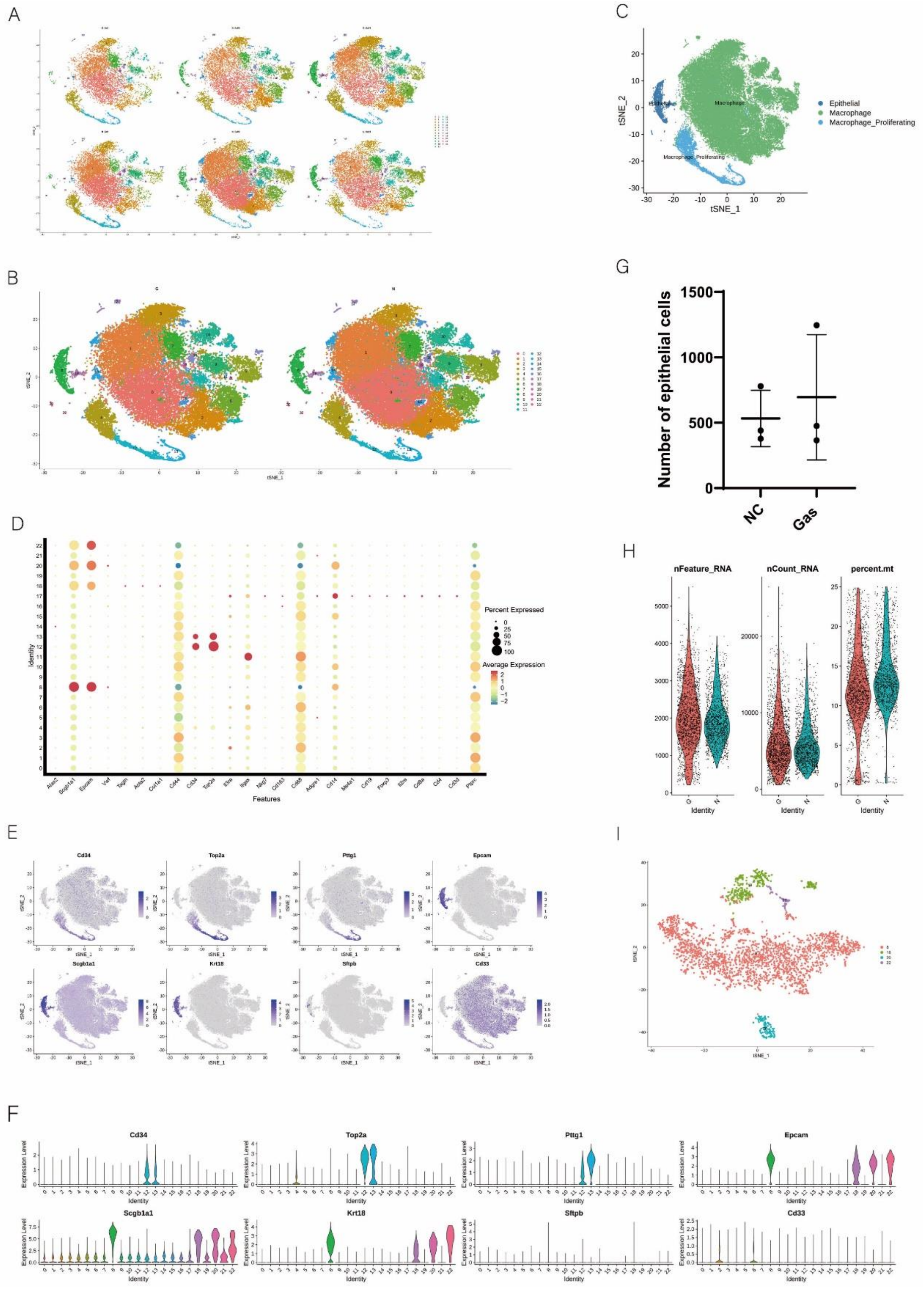

J

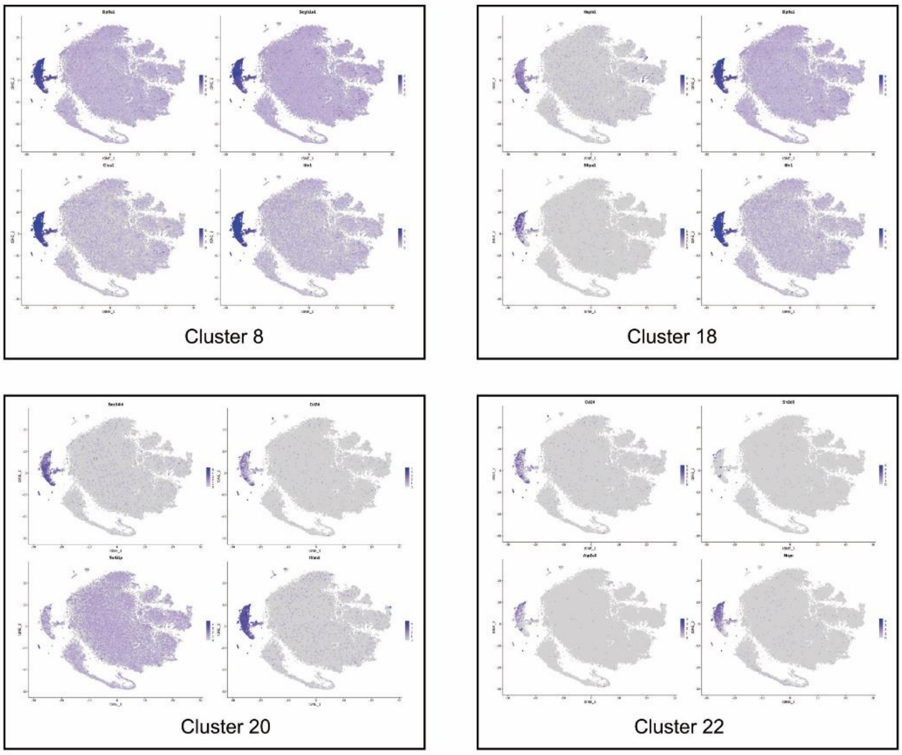
Cluster 8
Cluster 18
Cluster 20
Cluster 22

K

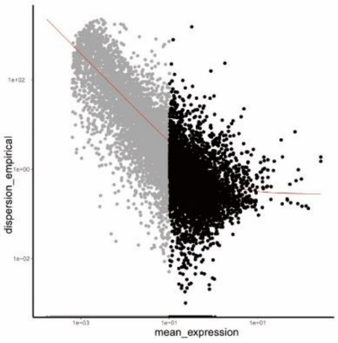
1e+02
1e+00
1e-02
dispersion_empirical
1e-03
1e-01
1e+01
mean_expression

L

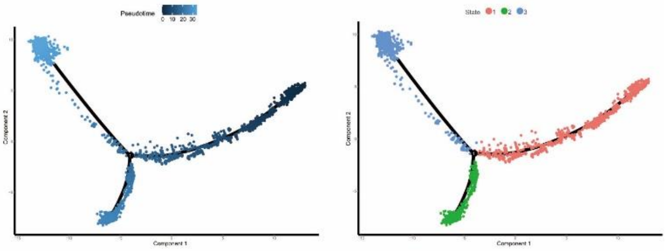
Pseudotime
0 10 20 30
Component 2
Component 1
State 1 2 3
Component 2
Component 1

Supplemental Figure S2

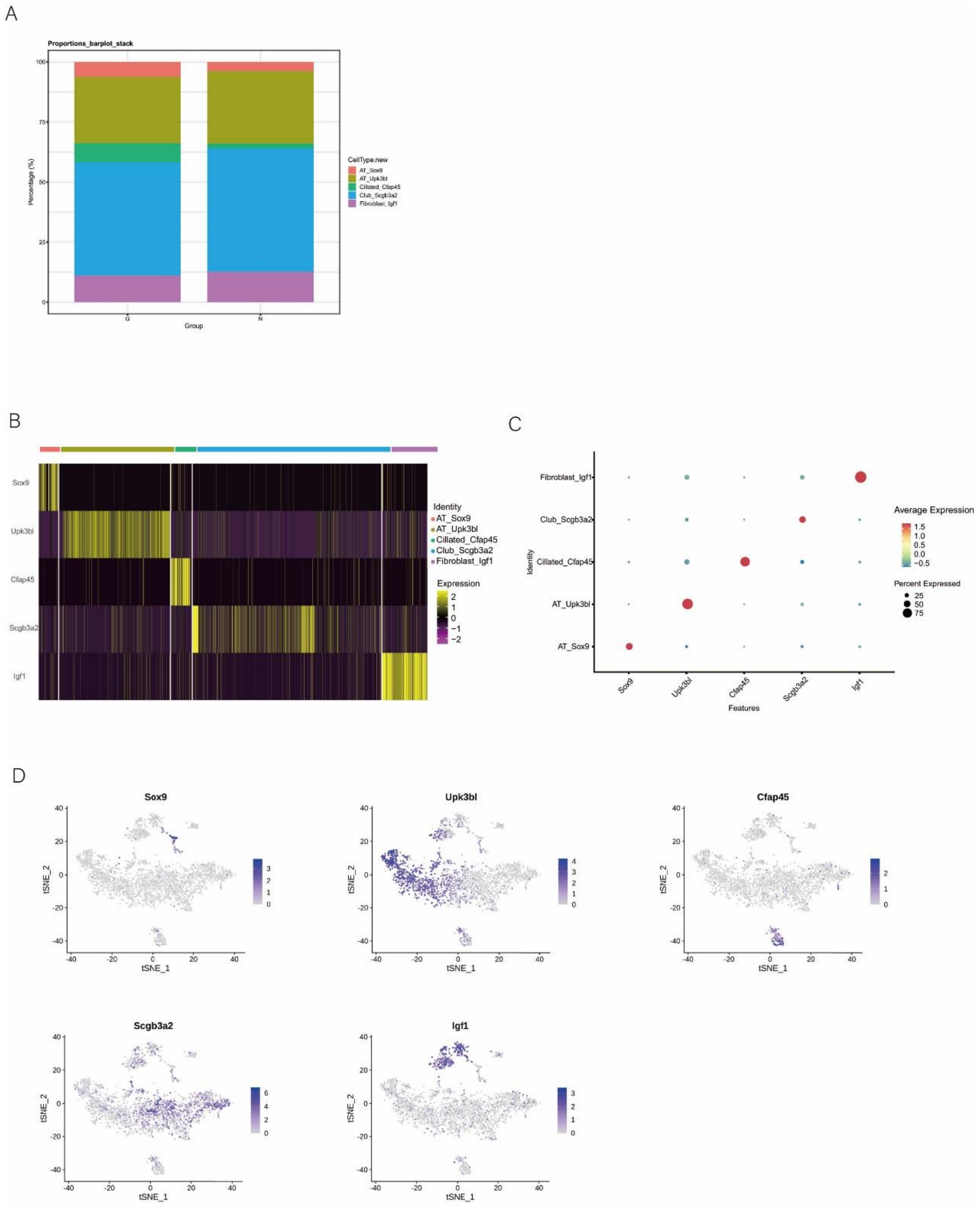

Supplemental Figure S3

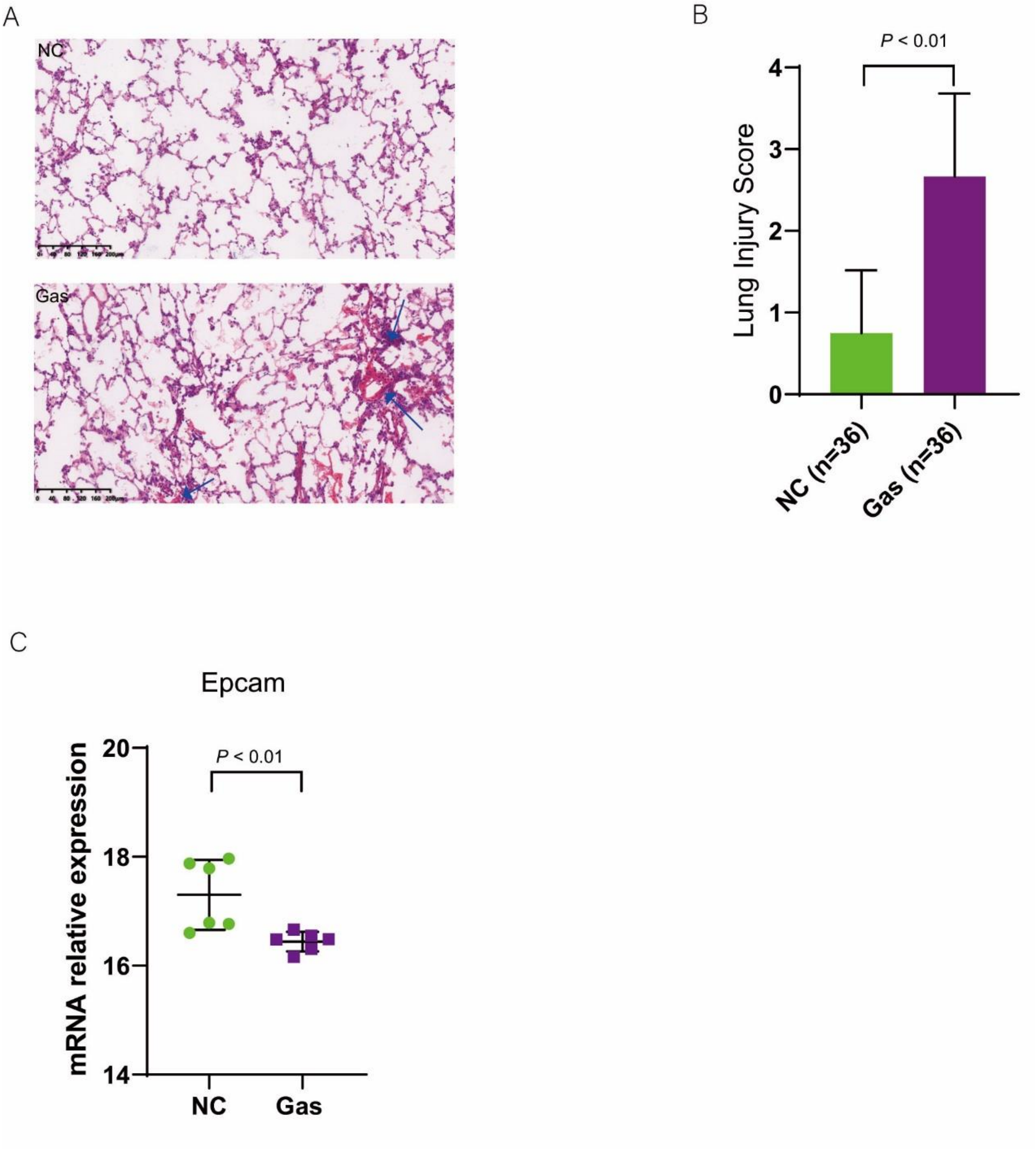

D

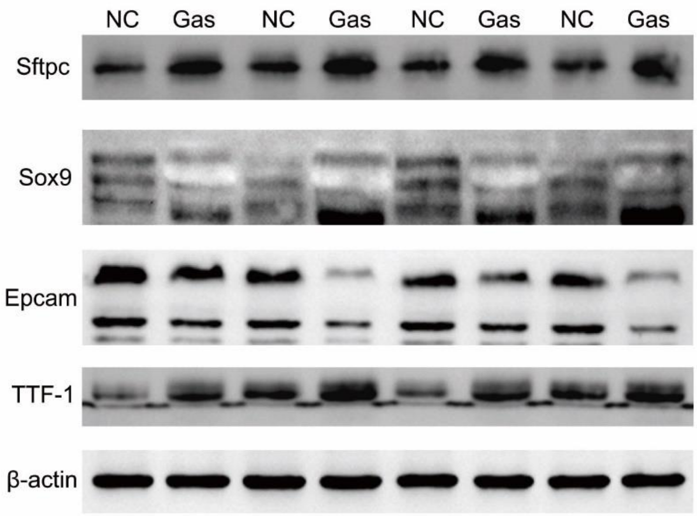
NC
Gas
NC
Gas
NC
Gas
NC
Gas
Sftpc
Sox9
Epcam
TTF-1
β-actin

E

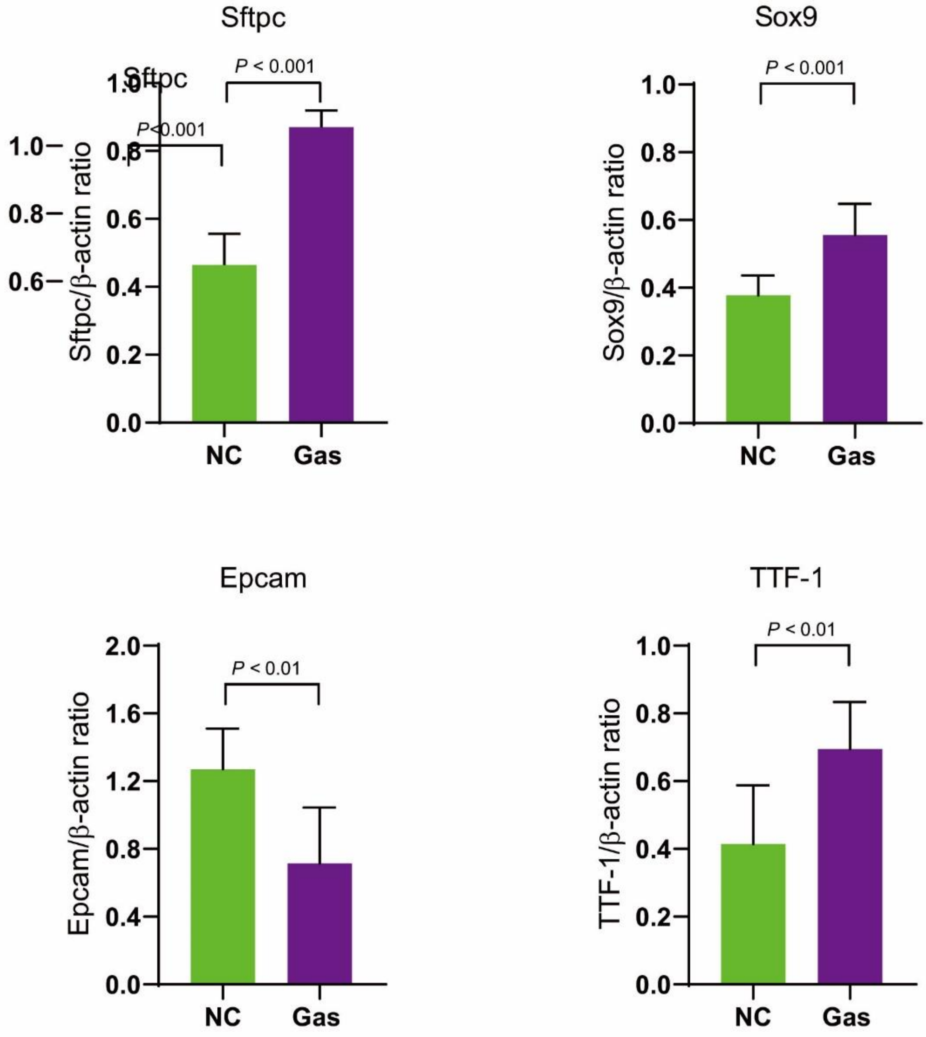
Sftpc
Sftpc
P < 0.001
P < 0.001
Sftpc/β-actin ratio
NC
Gas
Sox9
P < 0.001
Sox9/β-actin ratio
NC
Gas
Epcam
P < 0.01
Epcam/β-actin ratio
NC
Gas
TTF-1
P < 0.01
TTF-1/β-actin ratio
NC
Gas

Supplemental Figure S4

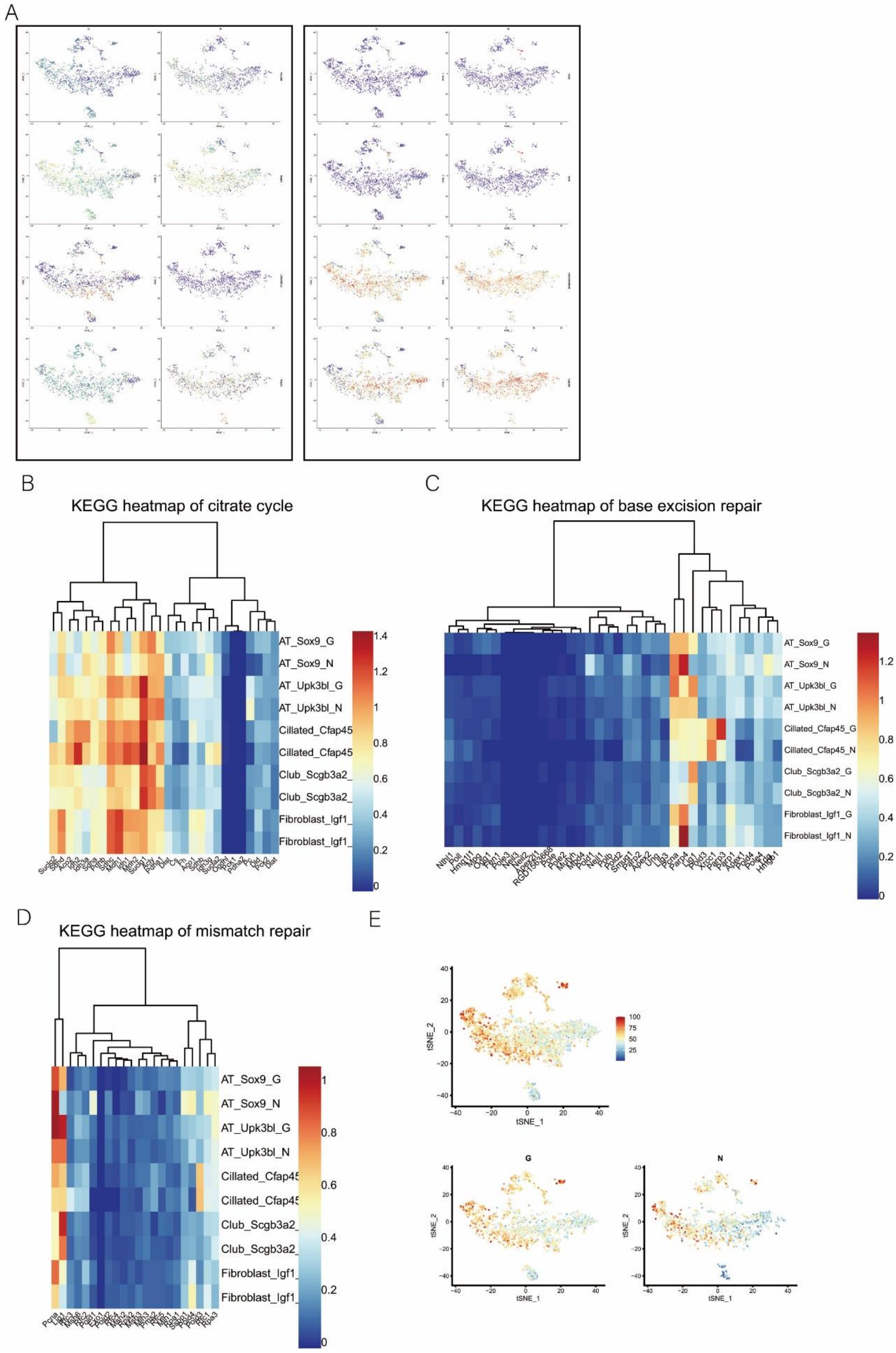

Supplement: Supplementary file 1 [file ijms-24-00277-s001.zip › ijms-1972690-supplementary.pdf]
